# A novel prognostic model for Hedgehog signaling pathway-related genes predicts the prognosis of hepatocellular carcinoma

**DOI:** 10.3389/fonc.2025.1674924

**Published:** 2026-01-13

**Authors:** Ting Liu, Yang Wang

**Affiliations:** 1 Department of Pathology, Beijing Ditan Hospital, Capital Medical University, Beijing, China; 2 Department of General Surgery, Beijing Ditan Hospital, Capital Medical University, Beijing, China

**Keywords:** HCC, hedgehog signaling pathway-related genes, prognosis, cox regression, TCGA, ICGC

## Abstract

**Background:**

The Hedgehog signaling pathway is involved in the carcinogenesis and development of hepatocellular carcinoma(HCC); however, few studies on the prognostic model of the Hedgehog signaling pathway have been found.

**Methods:**

First, we selected 56 Hedgehog signaling pathway-related genes from the UALCAN database and used the DESeq2 method to screen out differentially expressed genes in HCC and normal liver samples. Validation was performed using quantitative real-time PCR (qRT-PCR) of HCC and normal tissues. Second, the differentially expressed genes were analyzed using Cox and Lasso regression, and a five-gene prognostic risk model was established. Risk scores were divided into high-risk and low-risk groups, and their correlation with clinical indicators and immunity was analyzed. Univariate and multivariate regression analyses were performed for risk scores. Finally, a comparative functional enrichment analysis was conducted to delineate the biological pathway disparities between the high- and low-risk groups. The effectiveness of the model was validated using the ICGC database.

**Results:**

The prognostic risk model of five genes(BMP2,CSNK1D,CSNK1E, ZIC2, and PRKACB) was obtained after screening 56 Hedgehog signaling pathway-related genes. Risk scores emerged as robust independent predictors of overall survival through univariate and multivariate analyses, independent of other clinicopathological parameters, in TCGA and ICGC cohorts. A significant correlation was observed between risk scores and immune microenvironment characteristics, including immune cell infiltration and immune checkpoint expression levels. qRT-PCR analysis confirmed that the expression of the five genes in the prognostic model was higher in HCC tissues than in normal tissues. Moreover, some functional pathways were identified in the high-risk group in the low-risk group.

**Conclusions:**

We developed a novel five-gene prognostic model centered on Hedgehog signaling pathway-related genes, which holds potential as a valuable prognostic tool for HCC and may provide guidance for immunotherapy selection.

## Introduction

Primary liver cancer, a prevalent malignant tumor of the human digestive system, has shown increasing incidence in recent years. In China, the yearly mortality rate due to liver cancer exceeds 360,000 cases. Liver cancer is the sixth most commonly diagnosed cancer and the third most deadliest cancer worldwide. Hepatocellular carcinoma (HCC), the most prevalent form, accounts for 75%–85% of liver cancer cases (1). Primary liver cancer development is strongly linked to the overexpression of oncogenes and the inactivation of tumor suppressor genes (2). HCC tends to be found at a later stage in patients who are not being actively surveilled (3). However, the molecular mechanisms underlying HCC pathogenesis remain unclear. Identifying and screening gene functions are crucial in molecular biology and translational medicine research, aiding in understanding disease pathogenesis and guiding clinical treatment strategies (4). It is widely believed to be associated with abnormal activation or inhibition of multiple signaling pathways, such as the MAPK, Notch, Wnt, PI3K/AKT/mTOR, and Hedgehog signaling pathways.

The Hedgehog (Hh) pathway is an effective oncogenic driver signal that promotes tumor proliferation, survival, angiogenesis, invasion and metastasis, metabolic recombination, and epithelium-interstitial transition (2); however, its role in the regulation of anti-tumor immune responses has received attention in recent years. The Hh signaling pathway is activated via two pathways during the initiation and development of tumors: classical and non-classical. Typical Hh signaling pathways include Hh ligands (SHH, IHH, and DHH), PTCH receptors (PTCH1, PTCH2), SMO receptors, SUFU, and Gli transcription factors (Gli1, Gli2, and Gli3) (5, 6). The non-classical Hh signaling pathway is divided into three types: Type I is the PTCH-mediated Hh signaling pathway, independent of downstream Smo/Gli, and is further divided into two subtypes: Type IA and Type IB, among which type IA is a PTCH-mediated apoptosis independent of Smo/Gli, and type IB is a PTCH-mediated cell cycle regulation independent of Smo/Gli. Type II is the SMO-mediated Hh signaling pathway; Type III is Gli, which mediates the Hh signaling pathway and does not depend on any upstream Gli signals. In HCC, activation of the Hh pathway supports tumor cell growth and reduces apoptosis at different stages of liver cancer development (7). In liver cancer tissues and cell lines, the expression of important components of the Hh signaling pathway is increased, which is strongly associated with the malignant degree of liver cancer and lymph node metastasis (8, 9). Studies have indicated that inhibiting Gli1 can not only suppress liver cancer growth by blocking Hh signaling pathway, but also inhibit HCC migration and invasion through the downregulation of MMP-2 and MMP-9 and suppression of epithelial-mesenchymal transition (EMT). Therefore, inhibition or interference with Gli1 expression can inhibit tumor progression (10). Huang et al. demonstrated that the Hh signaling pathway is activated in approximately 50% of HCC (8). Liu et al. revealed that Shh signaling molecules are expressed in human HCC cells (BEL-7402, Huh7, and HepG2). Shh-blocking antibodies downregulate the expression of Shh signaling effectors Ptch, Glil, and Gli2, inhibit the growth of these HCC cells, and induce apoptosis (11). This result indicated that Hh signaling pathway is involved in HCC and inhibiting the activation of this pathway can effectively promote the apoptosis of HCC.

Our study aimed to assess the predictive value of Hedgehog signaling pathway-related genes in HCC. Using univariate Cox regression, LASSO regression, and multivariate Cox regression, we established a five-gene prognostic risk model (CSNK1D, CSNK1E, ZIC2, BMP2, and PRKACB) to predict outcomes in patients with HCC. The predictive accuracy of the model is validated using multiple datasets. Furthermore, we investigated the relationship between the risk model and immune cell infiltration, immune checkpoints, and tumor mutation burden, thereby providing a theoretical foundation for identifying HCC biomarkers and potential targets for cancer immunotherapy.

## Materials and methods

### Data collection and screen diferentially expressed genes (DEGs)

A total of 56 Hedgehog signaling pathway-related genes were curated from the UALCAN database (12), with corresponding RNAseq data expression profiles and clinical annotations obtained from TCGA database and extracted the data in TPM format. Differences in mRNA expression between hepatocellular carcinoma and normal liver tissues were investigated using the DESeq2 method.

### Screening prognostic gene, establishment and validation prognosis model

Univariate Cox proportional regression analysis was performed on the differentially expressed genes (DEGs) to identify potential prognostic biomarkers using “survival” R package. Further selection was performed using the Least Absolute Shrinkage and Selection Operator (LASSO) regression algorithm. Cross-validation was utilized to determine the penalty parameter lambda through “glmnet” R pacage. Genes showing strong correlations were excluded to decrease the complexity of the model. Through LASSO regression analysis, we constructed a five-gene prognostic model. The risk score was calculated using the formula: Risk Score = Σ (Expi × Coefi), where Coefi represents the risk coefficient and Expi indicates the expression level of each gene. Based on the median risk score threshold, the cohort was dichotomized into high- and low-risk subgroups for subsequent analysis. Kaplan-Meier survival curves were generated to compare clinical outcomes between risk stratifications. Comprehensive regression analyses, including univariate and multivariate analyses, were performed to determine the independent prognostic value of the risk score using “survival”, “rms” R package. To validate the prognostic capability of the derived risk score, ROC curves were generated and the AUC was calculated with the ‘survivalROC’ R package, thereby assessing the risk. Moreover, the ICGC dataset, which includes 232 HCC samples along with related clinical data, was acquired for validation purposes.

### Immunity analysis in both risk group

Based on the ssGSEA algorithm provided by the R package GSVA, the immune infiltration status was calculated using the markers of 24 types of immune cells provided in the literature to evaluate 24 immune cell infiltration levels in the tumor microenvironment (TME) (13, 14). We systematically evaluated the expression patterns of immune checkpoint molecules across risk groups.

### Functional enrichment analysis

We employed the limma package to evaluate gene expression disparities between high-risk and low-risk groups in TCGA cohort, followed by functional enrichment analysis using the ClusterProfiler package. We selected the KEGG set in the MSigDB collections for functional analysis. We considered P < 0.05 and FDR < 0.25 as the criteria for significance.

### Single-cell RNA-seq data analysis

TISCH, the Tumor Immune Single-cell Hub database (http://tisch.comp-genomics.org/), is a standardized repository designed for processing RNA-Seq data at the single-cell level using a visualization model. We utilized the TISCH online platform to perform standard scRNA-seq analysis on the GSE166635 dataset, which contains single-cell sequencing data from 22631 cells of 2 patients with primary HCC. Differential gene analysis was used to initially obtain Hedgehog-related genes.

### Validation of model gene expression in clinical samples

A total of 10 HCC patients were collected in the study from January 2024 year to Dec 2024. Cancer and paracancerous tissues from HCC patients were collected for real-time quantitative polymerase chain reaction (qRT-PCR). This study was approved by the Ethics Committee of Beijing Ditan Hospital (DTEC-KY2025-037-02). Written informed consent was obtained from all the participants. The Fast King cDNA first-strand synthesis kit (QIAGEN,Germany,ID:73504) was used for reverse transcription. The integrity and quality of RNA was evaluated by Agilent 2100 Bioanalyzer(Agilent Technology). Relative quantitative analysis of the data was carried out using the 2−^△△^CT method. Primers for qRT-PCR were designed using the primer-blast function at the National Center for Biotechnology Information and were synthesized by RiboBio Co., Ltd. Biotech (Guangzhou) as follows:

GAPDH-F1:TGACTTCAACAGCGACACCCA

GAPDH-R1:CACCCTGTTGCTGTAGCCAAA

BMP2-F1:GAAGGTTACTCTGGCAAAGTGCT

BMP2-R1:ATAATCTTGAAAGTCAGCAGAGTGG

CSNK1D-F1:GTCAAAACCAAACACCCTCAGC

CSNK1D-R1:CCATCACCATGACGTTGTAGTCC

CSNK1E-F1:AGTTCCCACCGAGTTTTCCG

CSNK1E-R1:GTCAGCCACTGTCATCTCAGAGT

PRKACB-F1:GCAGAACTTGGACATTATGTGGA

PRKACB-R1:CTTATTGTAGCCCTTGCTGAGAA

ZIC2-F1:CCAACTTCAATGAATGGTACGTG

ZIC2-R1:AACAACATGATCACAAGGTGCC

### Statistical analysis

The mRNA data of 56 genes for Hedgehog signaling pathway in the TCGA database were standardized and then analyzed for differences using DESeq2 to ensure the consistency of the results. Cox regression and LASSO analyses were applied for model construction using “survival” and “glmnet” in R package. Survival analysis utilized Kaplan-Meier estimation method and was assessed using the log-rank test. The ‘survival’ and ‘survminer’ R packages were utilized for conducting survival analysis and creating visualizations, whereas the ‘timeROC’ package was applied for time-dependent ROC analysis. Univariate regression and multivariate regression were used to identify prognostic factors predicting OS. To determine predictive accuracy and clinical utility, time-dependent ROC curves and calibration plots were used to assess model performance. Analysis of the qRT-PCR experiments was conducted using the t-test method. Statistical significance was set at a P-value <0.05.

## Results

### Screen the differentially expressed genes of hedgehog signaling pathway and related genes

**Figure 1:**
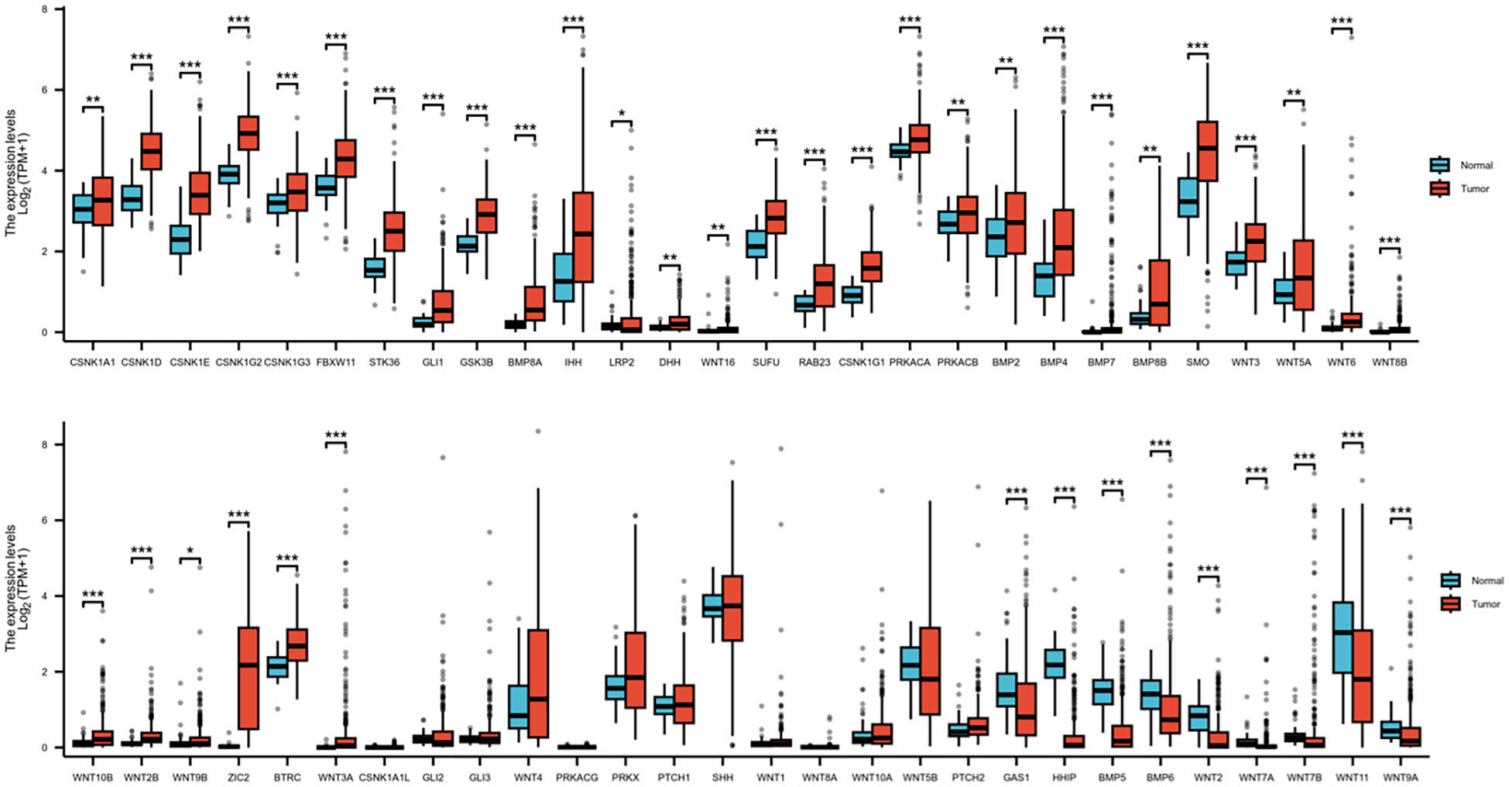
The differential expression of Hedgehog signaling pathway-related genes in HCC and normal tissues (***P<0.001,*P<0.01,*P<0.05).

According to the UALCAN database, there are 56 Hedgehog signaling pathway-related genes. We downloaded the expression profile data of 56 genes and used the DESeq2 method to investigate DEGs in 374 HCC and 50 normal liver tissues. The results showed that, in comparison to normal tissues, 34 genes were upregulated, 9 genes were downregulated, and 13 genes were not significantly altered (**Figure 1**).

### Establish a prognostic risk model of hedgehog signaling pathway-related genes

**Figure 2:**
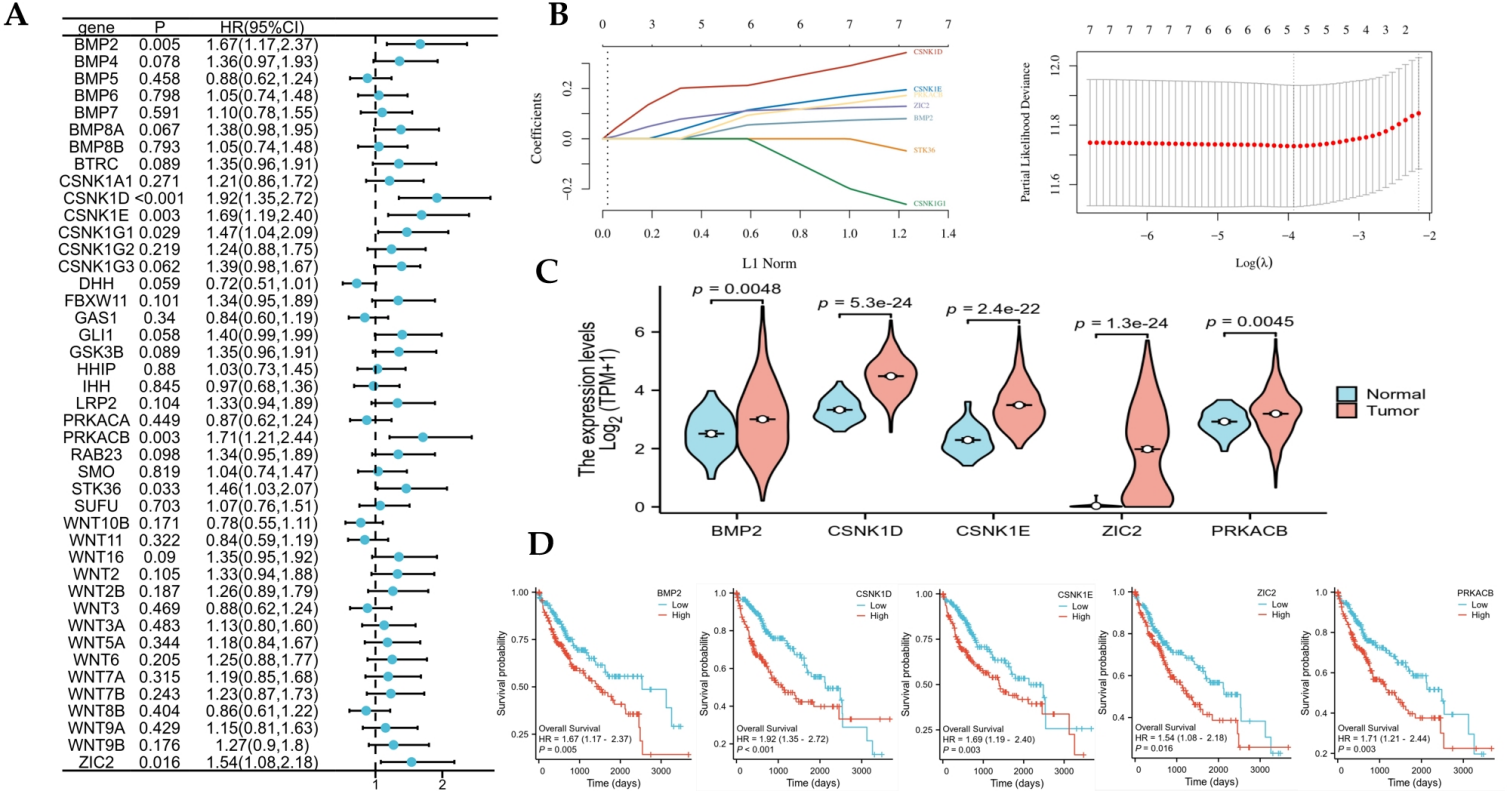
The prognostic significance of Hedgehog signaling pathway-related genes and the expression of five gene in hepatocellular carcinoma(HCC). **(A)** The Cox regression of differentially expressed genes in HCC. **(B)** The Lasso regression analysis of prognostic genes. **(C)** The differential expression of prognostic risk genes in HCC and normal tissues. **(D)** The Kaplan-Meier survival analysis of prognostic risk genes in HCC.

Cox regression analysis of 43 DEGs demonstrated that seven genes were related to prognosis: BMP2, CSNK1D, CSNK1E, CSNK1G1, PRKACB, STK36, and ZIC2 (**Figure 2A**). Using Lasso regression, we established the five-gene prognostic risk model. The Risk Score was calculated as follows: Risk Score=(0.0483)×BMP2+(0.2116)×CSNK1D+(0.1047)×CSNK1E+(0.0822)×PRKACB+(0.1077)×ZIC2 (**Figure 2B**). In HCC, the mRNA expression of BMP2, CSNK1D,CSNK1E, ZIC2, and PRKACB was higher than that in normal tissues (**Figure 2C**). According to K-M survival analysis, high levels of BMP2, CSNK1D, CSNK1E, ZIC2, and PRKACB predict a worse prognosis (**Figure 2D**).

### Development of a prognostic risk stratification model for hepatocellular carcinoma

**Figure 3:**
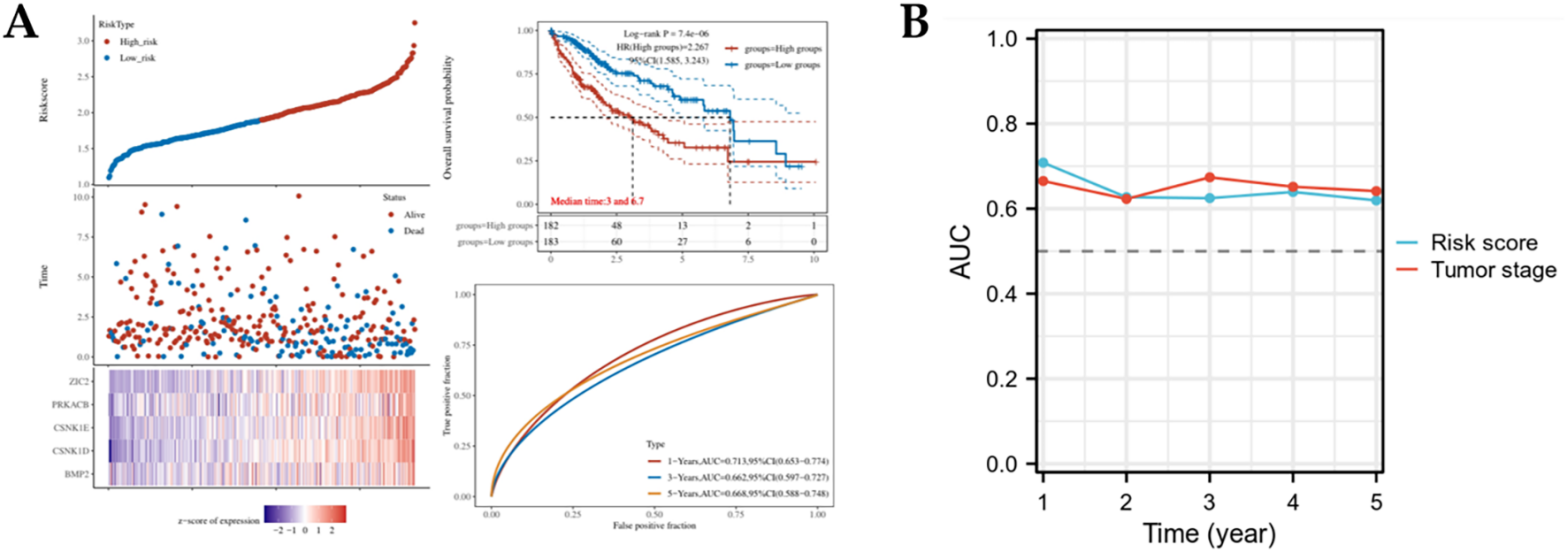
The predictive model for Hedgehog signaling pathway-based prognostic model in the TCGA databases. **(A)** Kaplan-Meier survival analysis and time-independent ROC of risk score in TCGA database. **(B)** The Comparison of risk scores and tumor stage in predicting the prognosis of HCC.

To evaluate the clinical applicability of the risk score-based prognostic stratification system, we stratified HCC patients into high- and low-risk cohorts using the optimal risk score as the cutoff value. Survival analysis revealed significantly poorer overall survival outcomes in high-risk patients than in low-risk patients. The TCGA cohort demonstrated 1-, 3-, and 5-year overall survival rates of 0.713, 0.662, and 0.668, respectively, further validating the prognostic stratification (**Figure 3A**). In addition, we compared the risk scores with the tumor stage to analyze the reliability of the model. The result demonstrated that compared with the tumor stage, the risk scoring model can better predict the prognosis of HCC (**Figure 3B**).

### Validation a prognostic risk model in HCC using ICGC database

**Figure 4:**
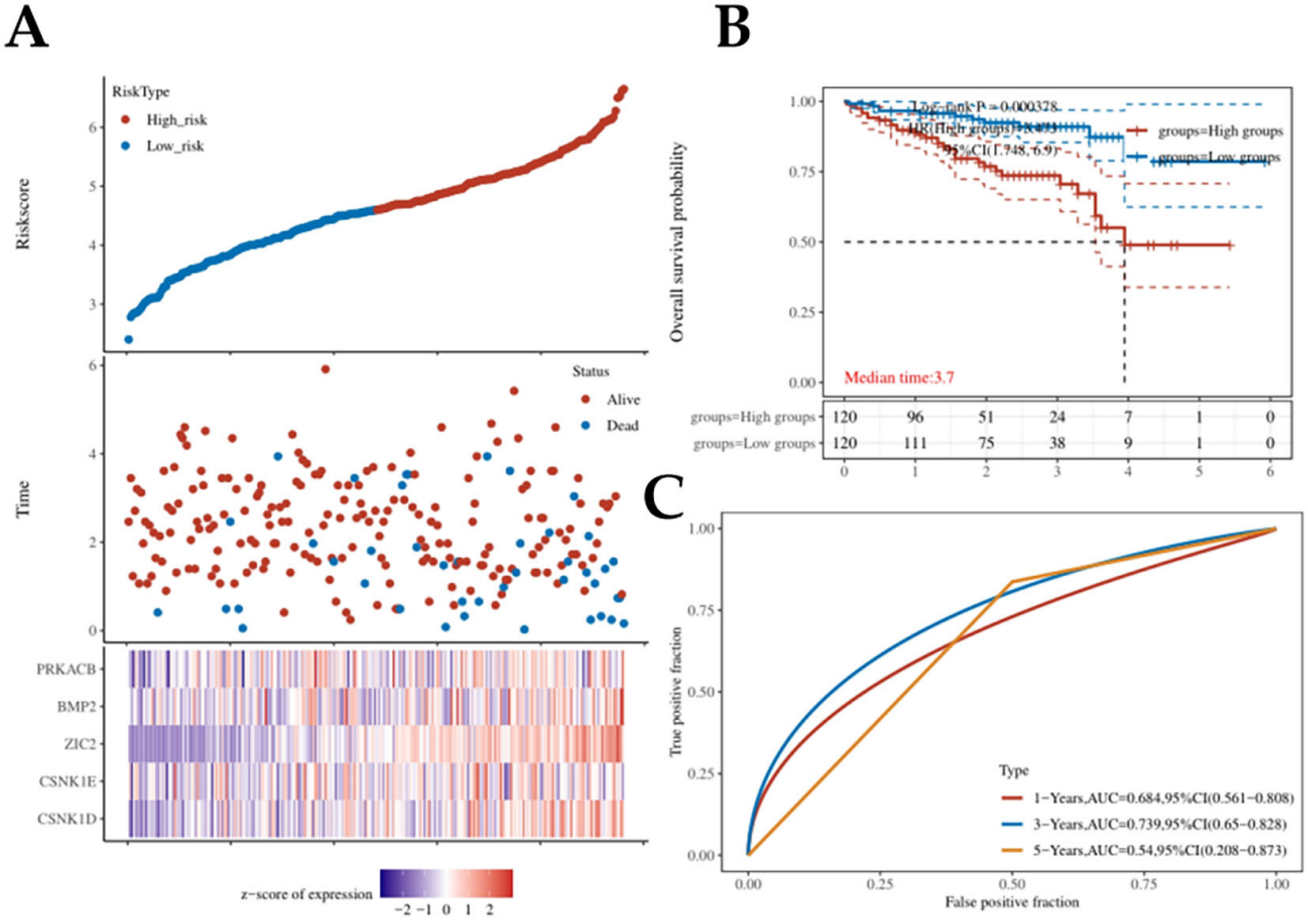
The validation of Hedgehog signaling pathway-related genes in HCC using ICGC database. **(A)** The scores for risk were separated into high-risk and low-risk categories. **(B)** Kaplan-Meier analysis of overall survival for two risk groups in ICGC database. **(C)** The 1-, 3-, and 5-year overall survival of patients in ICGC was predicted using time ROC curves.

To externally validate the prognostic model’s generalizability, we employed the ICGC cohort as an independent validation dataset. Consistent with our primary findings, the high-risk group exhibited significantly reduced overall survival compared to the low-risk group (p < 0.05). Overall survival rates at 1, 3, and 5 years were 0.684,0.739, and 0.54, respectively (**Figure 4**). These findings demonstratedf that the Hedgehog signaling pathway-based prognostic risk model serves as a robust predictive tool for clinical outcomes in HCC, potentially enabling more accurate risk stratification and personalized treatment strategies.

### Clinicopathological features and mutation analysis of the prognostic risk model of hedgehog signaling pathway-related genes in hepatocellular carcinoma

**Figure 5:**
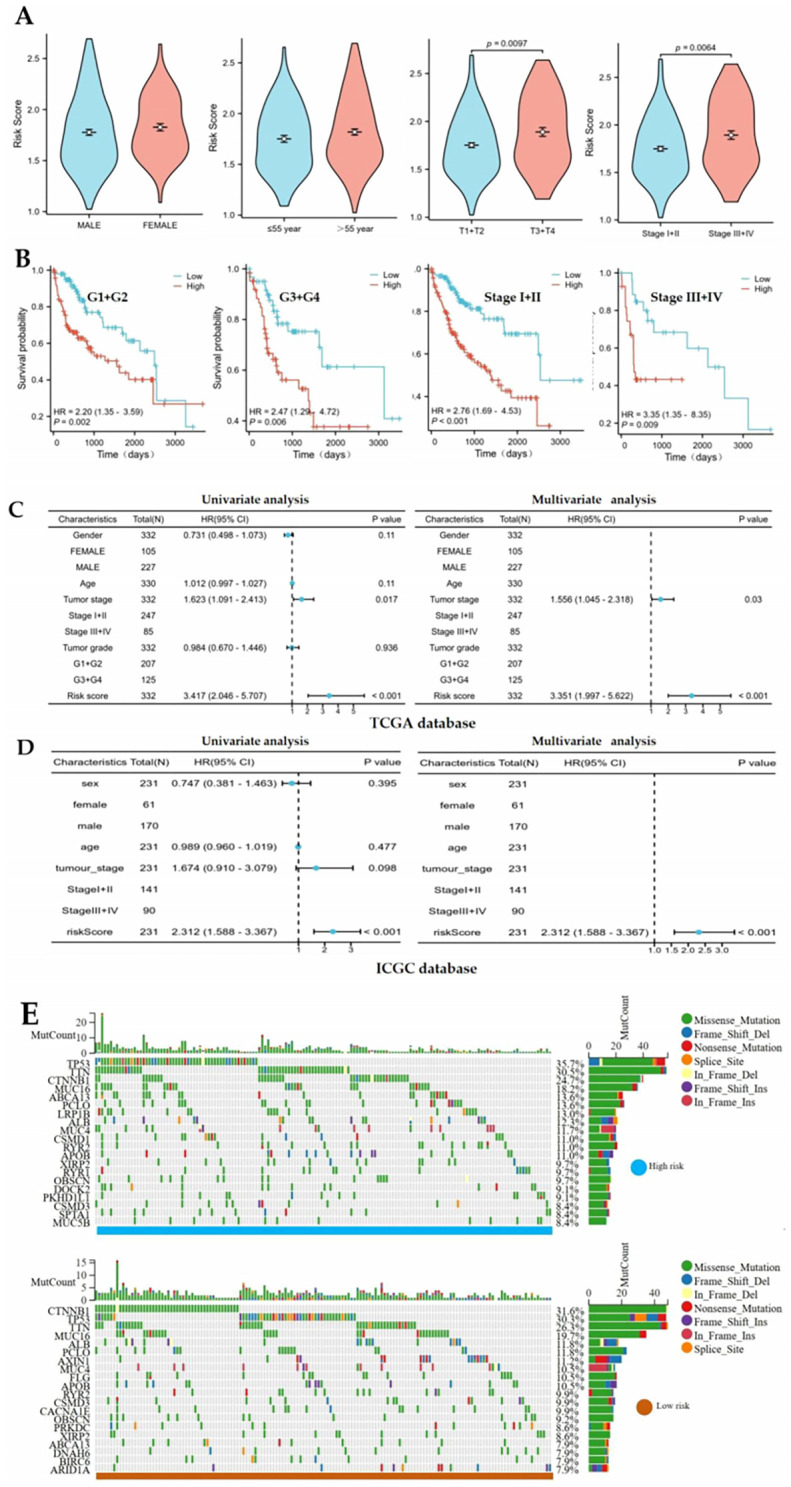
The association of risk score with clinical features and somatic mutation in HCC. **(A)** The differential analysis of risk score in sex, age, T stage and pathological stage. **(B)** Kaplan-Meier curve of both risk group in G1+G2 group, G3+G4 group, stage I+II group and stage III+IV group. **(C, D)** The prognostic significance of the risk score was consistently demonstrated as an prognostic factor in both TCGA (training) and ICGC (validation) cohorts. **(E)** Differential somatic mutation patterns between high-risk and low-risk groups.

This study comprehensively evaluated the prognostic value of the gene signature by investigating the association between the risk score and clinicopathological characteristics. Statistical analysis revealed a strong positive correlation between the risk score and disease progression, with significantly higher scores observed in advanced-stage tumors (T3-T4 vs T1-T2, Stage III-IV vs Stage I-II). However, the risk score showed no significant association with baseline characteristics, including age and sex (**Figure 5A**). Furthermore, survival analysis revealed significantly poorer overall survival in high-risk patients across histological grades and clinical stages. Specifically, this survival disparity was consistently observed in both well-differentiated tumors (G1+G2) and poorly differentiated tumors (G3+G4), as well as in early stage (Stage I-II) and advanced-stage (Stage III-IV) disease (**Figure 5B**). To evaluate the independent prognostic value of the risk score, we conducted Cox proportional hazards regression analyses adjusting for key clinical variables, including sex, age, tumor stage, and histological grade. The risk score emerged as a significant independent predictor in both the univariate and multivariate analyses, confirming its robust prognostic utility (**Figure 5C**). Validation of the ICGC-independent cohort confirmed that the risk score was a robust independent prognostic factor (**Figure 5D**). Comparative genomic analysis revealed distinct somatic mutation profiles between the risk groups. Although missense mutations represented the predominant variant classification in both cohorts, the high-risk group exhibited a characteristic mutation signature dominated by TP53, TTN, and CTNNB1. Conversely, the low-risk group demonstrated a different mutational hierarchy, with CTNNB1 mutations being the most prevalent, followed by TP53 and TTN, suggesting potential molecular mechanisms underlying differential clinical outcomes (**Figure 5E**).

### Comprehensive analysis of immune microenvironment features associated with the prognostic risk model in hepatocellular carcinoma: immune cell infiltration and checkpoint expression profiles

**Figure 6:**
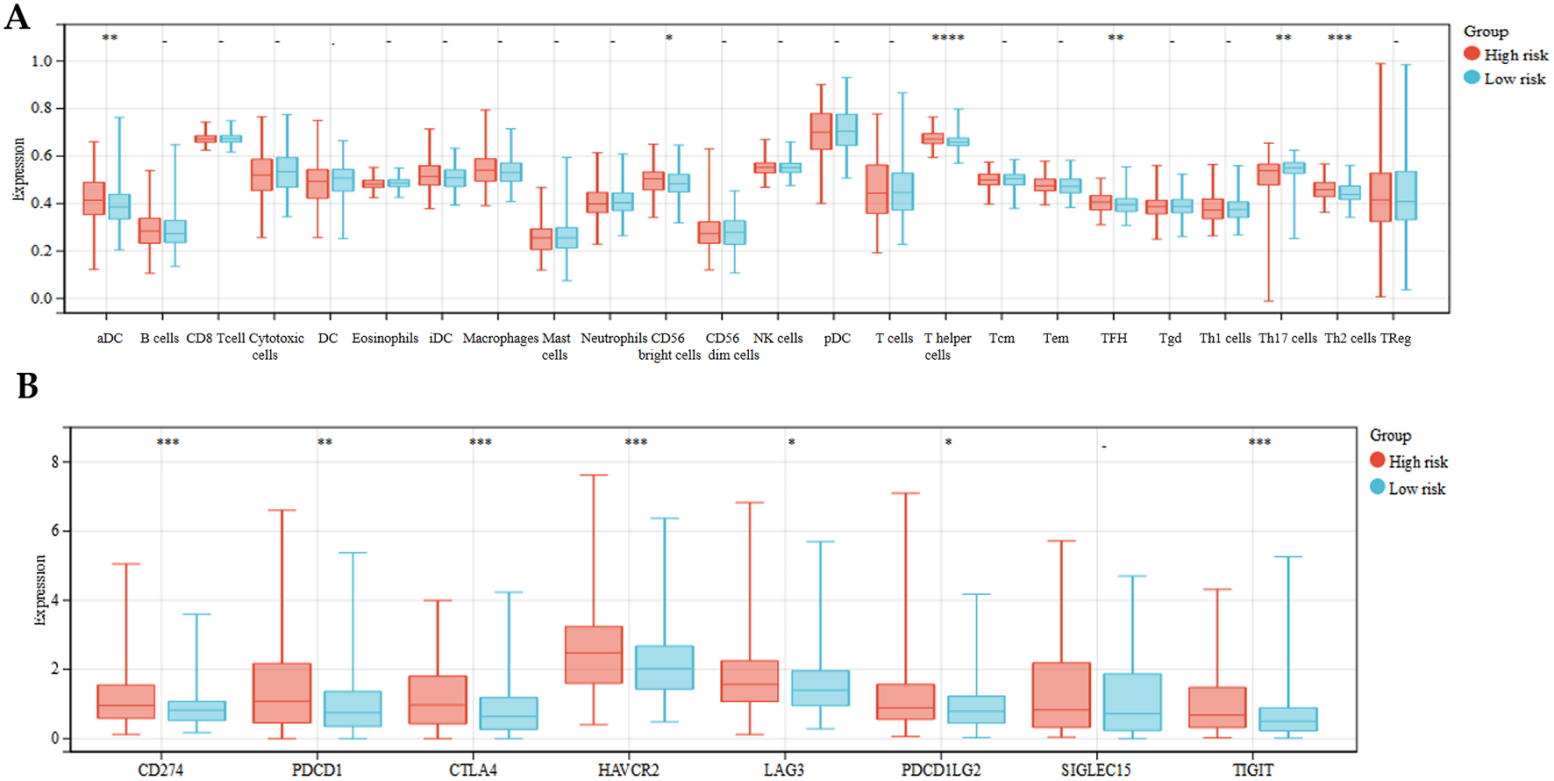
Distinct immune landscape features between risk groups. **(A)** ssGSEA-based quantification of immune cell infiltration scores in low-risk and high-risk subgroups. **(B)** Comparative analysis of immune checkpoints expression levels across risk stratifications.(****P<0.0001, ***P<0.001, **P<0.01, *P<0.05).

The Tumor Microenvironment (TME) constitutes a sophisticated biological niche encompassing heterogeneous cell populations, extracellular matrix components, molecular signaling networks, and unique biophysical properties. This intricate system significantly influences fundamental cancer processes, including tumorigenesis, progression to metastasis, and treatment outcomes, thereby serving as a crucial determinant of both cancer biology and therapeutic interventions. To systematically evaluate tumor microenvironment characteristics, we employed the single-sample Gene Set Enrichment Analysis (ssGSEA) algorithm to quantify associations between risk scores and immune landscape features, encompassing 24 distinct immune cell infiltration phenotypes. The results suggested that the expression of Th17 cells was higher in the low-risk group, whereas in the high-risk group, aDC, CD56 bright cells, T helper cells, TFH, Th2 cells was higher (**Figure 6A**). Immune checkpoints are a class of regulatory molecules in the immune system responsible for maintaining the balance of the immune response and preventing autoimmune diseases caused by excessive immune response. However, tumor cells often use these checkpoint molecules to evade immune system attack. Therapies targeting immune checkpoints, such as immune checkpoint inhibitors, have become important breakthroughs in cancer treatment. Our results showed that the expression of CD274(PD-L1), PDCD1(PD-1), CTLA4, HAVCR2, LAG3, PDCD1LG2, and TIGIT was higher in the high-expression group than in the low-expression group (**Figure 6B**).

### The functional enrichment analysis of risk score

**Figure 7:**
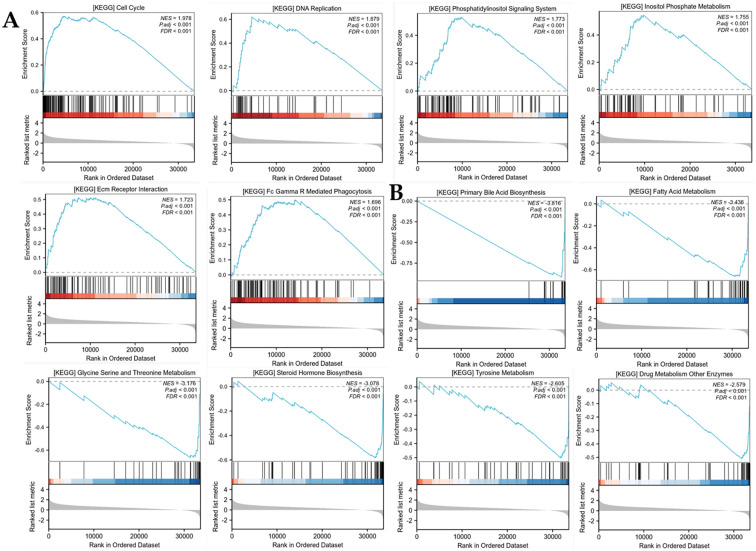
Comprehensive pathway analysis using GSEA. **(A)** The GSEA in the high-risk cohort. **(B)** The GSEA in the low-risk cohort.

Patients were stratified into low- and high-risk groups based on the median risk score cutoff. Gene Set Enrichment Analysis (GSEA) was employed to elucidate the biological pathways and molecular mechanisms related to the high-risk phenotype, revealing distinct pathway activation patterns characteristic of aggressive disease biology. Pathway analysis in the high-risk cohort demonstrated predominant activation of cell pathways, including the cell cycle, DNAreplication, phosphatidylinositol signaling system, inositol phosphate metabolism, ECM receptor interaction, and FC-gamma-R-mediated phagocytosis (**Figure 7A**). The low-risk group was enriched in the following pathways, including primary bile acid biosynthesis, fatty acid metabolism, glycine serine and threonine metabolism, steroid hormone biosynthess, tyrosine metabolism and drug metabolism other enzymes (**Figure 7B**).

### ScRNA-seq analysis of five prognostic genes

**Figure 8:**
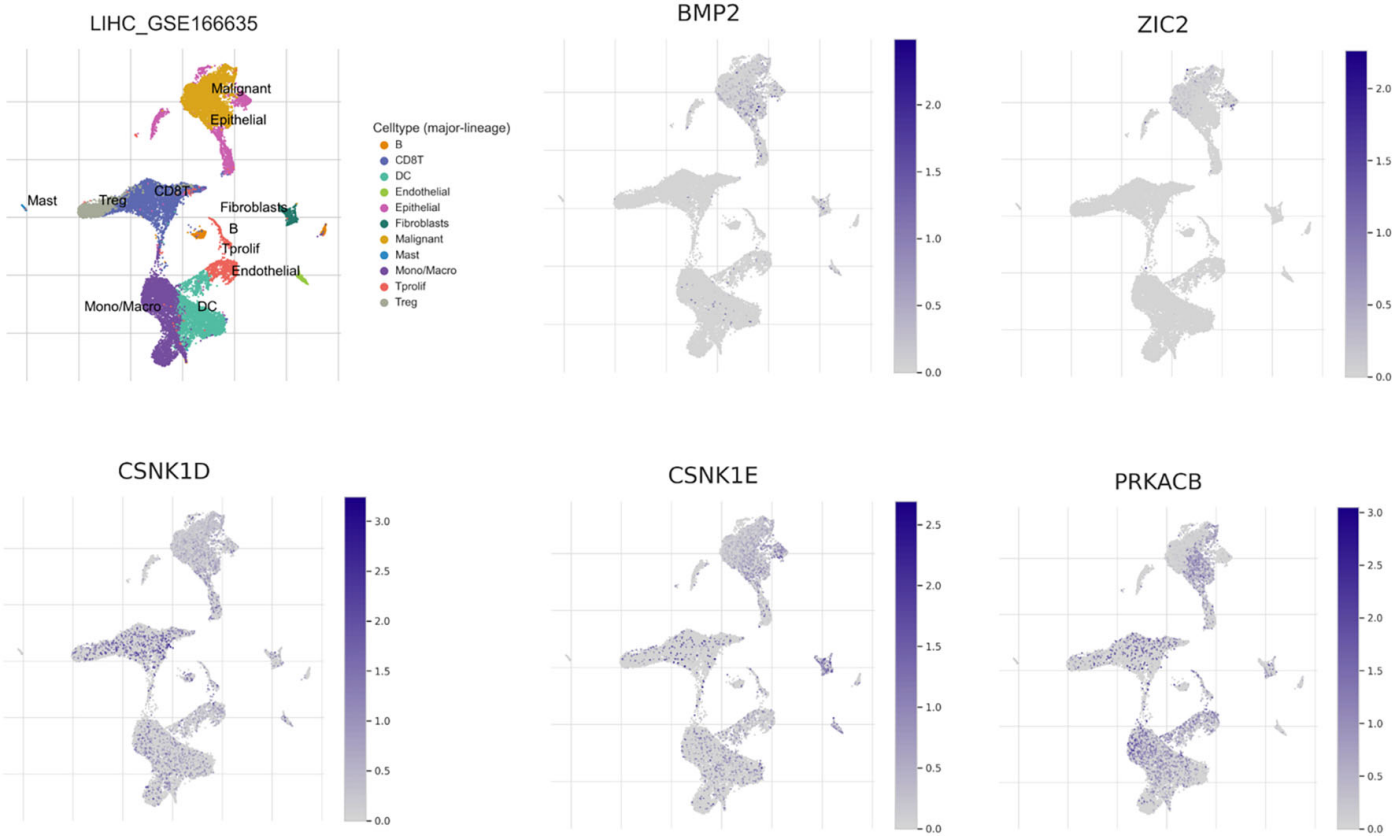
Single cell RNA sequencing analysis of five prognostic genes.

We utilized the TISCH 2.0 database to conduct scRNA-seq analysis, aiming to investigate the function of five prognostic genes in HCC at the single-cell level. We chose GSE166635 to discuss the expression of five genes at the single-cell level. GSE166635 contained two patients and 22631 cells. GSE166635 mainly analyzed the malignant cells, epithelial cells and immune cells. In malignant cells, BMP2, ZIC2, CSNK1D, CSNK1E, and PRKACB showed elevated expression levels. Additionally, BMP2, CSNK1D, CSNK1E, and PRKACB were significantly more expressed in immune cells (**Figure 8**).

### Verification of BMP2, CSNK1D,CSNK1E,PRKACB,ZIC2 expression

**Figure 9:**
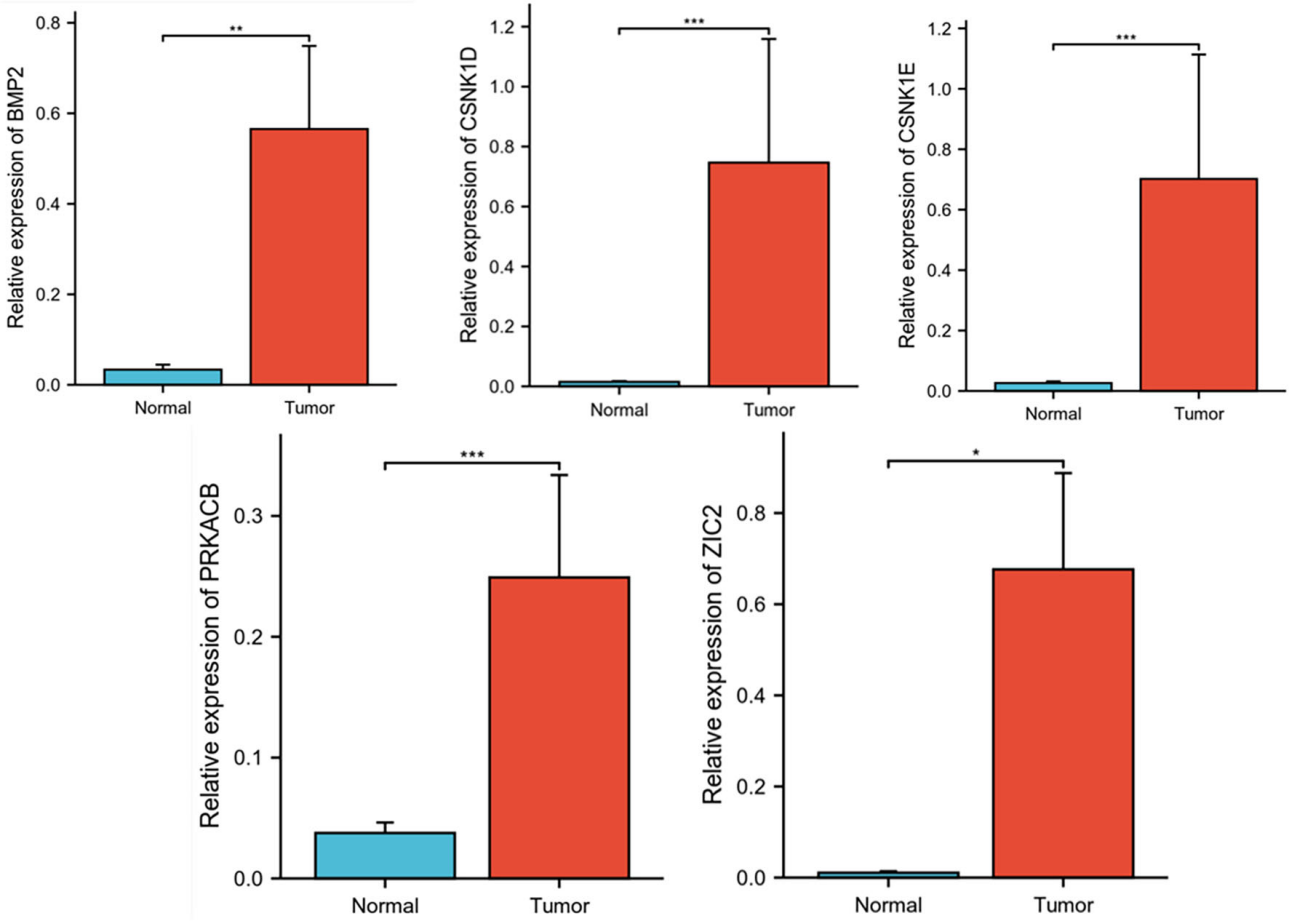
Verification of BMP2, CSNK1D, CSNK1E, PRKACB and ZIC2 expression by qRT-PCR. (***P<0.001, **P<0.01, *P<0.05).

The expression of the model genes BMP2, CSNK1D, CSNK1E,PRKACB, and ZIC2 was verified using qRT-PCR. As shown in **Figure 9**, the results indicated that the expression trends of the model genes were consistent with those of the TCGA-HCC dataset.

## Discussion

The Hedgehog (Hh) signaling pathway, alternatively referred to as the Hh-Ptch, Hh-Gli, or Hh-Ptch-Smo pathway, represents an evolutionarily conserved mechanism for transmitting signals from the cell membrane to the nucleus. This pathway is critically involved in regulating normal embryonic development across invertebrate and vertebrate species (15). Recent studies have found that the Hh signaling pathway is abnormally activated in a variety of malignant tumors, and its abnormal activation can promote the proliferation, invasion, migration, and multi-drug resistance of tumor cells (6). Abnormal activation of Hh signalling may accelerate the growth of liver cancer cells. Cross-talk between Hh signaling and the TME affects tumor growth, immune tolerance, inflammation, and drug resistance (16). However, the development and validation of Hedgehog pathway-related prognostic models for HCC remains unexplored.

There are many reported gene signatures for HCC. Chengbin Guo found a novel NK cell-related signature. The signature can effectively predict the prognosis of patients with HCC as well as the efficacy of immunotherapy (17). Tang et al. established a novel tumor immunological phenotype-related gene index (TIPRGPI) consisting of 11 genes, which is used to predict HCC prognosis and immunotherapy response (18). Studies have identified subtypes of HCC through genes related to cellular senescence (CSGs), and have constructed a subtype prediction model for cellular senescence-related genes and a new prognostic scoring system, with the aim of predicting the clinical outcomes 7and treatment responses of HCC (19). Qiu et al. had identified new characteristics of peroxisome-related genes, which may help improve the personalized prognosis prediction for patients with HCC (20). Li et al. established the CpG island methylator phenotype (CIMP) model to predict the prognosis of HCC (21). These various types of predictive models can all effectively predict the prognosis of HCC. Most studies have shown that the hedgehog signaling pathway is associated with HCC. We have established a prediction model for genes related to the hedgehog signaling pathway to predict the prognosis of HCC. In our study, 56 Hedgehog signaling pathway-related genes were used for research, and 43 differentially expressed genes were screened. Prognostic analysis of these 43 genes revealed that seven genes were significantly associated with overall survival outcomes. Prognostic risk models were established for five genes (CSNK1D,CSNK1E, ZIC2, BMP2, and PRKACB) after LASSO regression of the seven prognostic genes. We compared the clinicopathological and prognostic analyses between the two groups. Kaplan-Meier survival analysis demonstrated significantly reduced overall survival in high-risk patients compared with their low-risk counterparts. Furthermore, significant positive correlations between risk scores and both clinical T stage and pathological stage were observed, indicating its potential utility in tumor staging and disease progression assessment. In univariate and multivariate regression analyses, the risk scores were prognostic factors in both TCGA and ICGC. Validation in both TCGA and ICGC cohorts demonstrated robust predictive performance of the prognostic model, with area under the curve (AUC) values exceeding 0.5 for 1-year, 3-year, and 5-year survival predictions, confirming the model’s excellent prognostic accuracy in HCC. Moreover, compare with tumor stage, risk score had superior predictive accuracy for predicting HCC prognosis. In the present study, we used qRT-PCR to verify the differential expression of the five genes in HCC and normal tissues. This study revealed elevated expression levels of BMP2, CSNK1D, CSNK1E, ZIC2, and PRKACB in HCC tissues relative to normal controls, supporting the potential utility of these five genes in developing a robust HCC risk assessment model.

CSNK1D (CK1), a member of the CK1 family, was first identified and isolated by Graves et al. in the early 1990s. Its gene is located on the long arm of human chromosome 17, specifically at the 17q25.3 locus (22). In medulloblastoma, knocking out CSNK1D is sufficient to reduce the expression of HH target genes and GLI1 protein. This indicates that CSNK1D is essential for the activation of the classical HH signaling pathway and HH target genes (23). CSNK1D is upregulated in hepatocellular carcinoma and is associated with poor prognosis (24). As a prominent member of the bone morphogenetic protein (BMP) family, BMP-2 has been demonstrated to significantly potentiates tumor-associated angiogenesis by enhancing endothelial cell proliferation and migration, thereby facilitating robust vascular network formation within the tumor microenvironment. BMP-2 enhances vascularization and contributes to tumor angiogenesis by activating the Id1 and p38 MAPK signaling pathways (25). As previously reported, BMP2 exhibits abnormal upregulation in cancer stem cells (CSCs) in prostate cancer. Furthermore, emerging evidence demonstrates that BMP2 significantly contributes to HCC progression by promoting malignant phenotypes, including enhanced cellular proliferation, invasion, and metastasis, while simultaneously stimulating tumor angiogenesis and microenvironment remodeling. It was reported that silencing BMP2 significantly suppressed the proliferation, migration, and invasion of liver cancer cells *in vitro*. *In vivo* studies further revealed elevated BMP2 expression in HCC tissues, which facilitates angiogenesis and promotes HCC progression (26). ZIC proteins play essential roles in vertebrate embryonic development and the pathogenesis of human cancers (27). Under normal physiological conditions, ZIC2 expression is restricted to the brain and testis; however, its expression is dysregulated and detectable in multiple tumor tissues. Research has shown that ZIC2 expression gradually increases from normal to cancerous to metastatic tissues. ZIC2 expression was significantly positively correlated with PAK4 levels and associated with poorer overall survival and disease-free survival rates. ZIC2 promotes tumor growth and metastasis via PAK4 in HCC (28). Elevated expression levels of CSNK1E protein have been observed in multiple cancer types (29–31). The core signal transduction process of the Hedgehog pathway involves ligand binding to the Ptch receptor, which releases the inhibition of the Smo protein. The activated Smo then triggers downstream intracellular signaling cascades. CSNK1E acts downstream of Smo signaling, influencing the functional state of the Gli protein. Existing evidence indicates that CK1 isoforms, including CSNK1E, exhibit oncogenic properties through multiple mechanisms, including promotion of cellular proliferation, induction of genomic instability, and suppression of apoptotic pathways (32). Accumulating evidence from recent studies indicate that PRKACB plays a significant role in the oncogenic processes of various malignant tumors across multiple systems. Research has demonstrated that antisense oligodeoxynucleotides targeting protein kinase subunits can effectively induce growth inhibition, apoptosis, and cellular differentiation in diverse cancer cell lines, both in laboratory settings (*in vitro*) and living organisms (*in vivo*) *(*33). CSNK1D/E is responsible for integrating the biological clock and signal transduction. ZIC2 and GLI form a transcriptional complex to control the output of functions. BMP2 acts as an effector molecule and participates in positive feedback, while PRKACB may undergo oncogenic functional transformation. Each of them performs its own specific role and cooperates with each other to jointly form a powerful oncogenic module.

The cellular components of the tumor microenvironment (TME), particularly immune and stromal cells, play pivotal roles in modulating critical oncogenic processes, including tumor proliferation, invasive capacity, and metastatic dissemination through complex cell-cell interactions and paracrine signaling mechanisms (34). Moreover, immune and stromal cells in the TME can promote or inhibit programmed cell death of tumor cells by releasing cytokines and regulating cell signaling pathways (35). Consequently, we systematically evaluated the tumor immune microenvironment characteristics, including quantitative differences in immune cell infiltration and comparative analysis of immune checkpoint molecule expression between high- and low-risk cohorts. Our results showed that aDC, CD56 bright cells, T helper cells, TFH, Th2 cells were highly expressed in the high-risk group. These findings suggest that the prognostic model developed based on Hedgehog signaling pathway-related genes is closely linked to immune cell infiltration in HCC. Recent research has demonstrated significant advancements in tumor immunotherapy by targeting immune checkpoints, establishing it as a highly effective approach in cancer treatment. The high-risk group exhibited markedly enhanced immune checkpoint expression profiles, indicating potential susceptibility to immune checkpoint inhibitors and providing a rationale for targeted immunotherapy in this patient subset. Upregulation of immune checkpoint pathways in the TME creates an immunosuppressive niche that enables tumor cells to evade immune surveillance, ultimately leading to enhanced tumor proliferation, metastatic potential, and resistance to conventional therapies (36). Moreover, comprehensive genomic analysis of somatic mutation landscapes revealed distinct patterns between risk subgroups, with the high-risk group demonstrating a significantly elevated TP53 mutation frequency. The significantly lower TP53 mutation frequency observed in the low-risk cohort may underlie their favorable prognosis, as intact TP53 function preserves critical cellular processes, including genomic integrity maintenance, cell cycle arrest, and programmed cell death, thereby limiting tumor progression and enhancing treatment responsiveness.

To elucidate the molecular mechanisms mediated by Hedgehog signaling pathway-associated genes, we performed a comprehensive GSEA to distinguish significantly enriched bioprocesses, molecular functions, and signaling networks. We discovered that the high-risk group was related to signaling pathways, such as cell cycle, DNA replication, phosphatidylinositol signaling system, inositol phosphate metabolism, ECM receptor interaction, and FC-gamma-R-mediated phagocytosis. The low-risk group had participation in the pathways, such as primary bile acid biosynthesis, fatty acid metabolism, glycine serine and threonine metabolism, steroid hormone biosynthess, tyrosine metabolism and drug metabolism other enzymes. These pathways may lead to the progression of liver cancer in the high-risk and low-risk groups. Additionally, single-cell analysis demonstrated that the five genes had high expression in malignant cells. The findings suggested that high expression of five genes participated in the progression of HCC.

Importantly, although the five-gene signature showed excellent prognostic performance in TCGA dataset, its generalizability across diverse clinical settings requires further validation. The limitation is that the sample size is relatively small, validation in larger clinical cohorts is necessary for future clinical translation. In the future, we expanded multi-center prospective samples or conducting functional experiments to verify the model’s biological basis. In addition, although this predictive model is related to immune cells and immune checkpoints, its predictive value in terms of immune therapy response will serve as a potential indicator for subsequent research. These findings require validation using targeted biological experiments.

## Conclusion

In summary, our study identified five key prognostic genes, CSNK1D, CSNK1E, ZIC2, BMP2, and PRKACB, that exhibited significant differences in both risk groups. We established a risk prediction model that is independent of clinical factors and demonstrated strong performance in the robust stratification of patient risk stratifications across multiple validation datasets. Notably, the model represents a novel risk assessment tool for patients with HCC based on the Hedgehog signaling pathway and related gene signature.

## Data Availability

The datasets presented in this study can be found in online repositories. The names of the repository/repositories and accession number(s) can be found in the article/supplementary material.
